# Allergen challenge tests in allergen immunotherapy: State of the art 

**DOI:** 10.5414/ALX02322E

**Published:** 2023-03-01

**Authors:** Petra Zieglmayer, René Zieglmayer, Patrick Lemell

**Affiliations:** 1 Karl Landsteiner University, Krems, and; 2 Vienna Challenge Chamber, Vienna, Austria

**Keywords:** conjunctival, bronchial, nasal, allergen immunotherapy, challenge test

## Abstract

Introduction: Treatment effects in allergen immunotherapy (AIT) studies are based on symptomatic improvement, and evaluations of naturally exposed patients do often show weak efficacy. Allergen challenge tests, such as conjunctival (CAC), nasal (NAC), or bronchial (BAC) challenge tests, or challenges in allergen exposure chambers (AEC) are accepted by regulators for AIT phase II studies only. Materials and methods: This review aims to describe different allergen challenge test methods, summarizes safety and limitations for each, and discusses their potential for use in AIT trials. Results: Organ-specific allergen challenges provide information about individual reactivity, reaction threshold, and organ-specific efficacy of AIT. AECs, targeting all affected organs simultaneously, were developed to investigate disease mechanisms and treatment effects under controlled and reproducible conditions. Conclusion: A high level of standardization is existing for NAC only; in CAC and BAC, the toolbox is limited to subjective symptom scoring with no validated objective parameters identified yet. AECs are complex and heterogenous; correlation of systems and comparability of study data is claimed. All challenge methods are safe when conducted by experienced staff.

## Introduction 

Allergen-specific immunotherapy was first described more than 100 years ago and is considered the only treatment of airway allergies with persistent efficacy by induction of clinical and immunological tolerance. Until today, treatment success is limited by treatment-related adverse reactions, so novel approaches mainly target improvement of tolerability apart from optimized immunogenicity. Several innovations, such as synthetic vaccine development programs or antibody-derived passive vaccination and an increasing amount of non-injectable extract-based formulations, are currently evaluated in clinical trials. According to regulatory requirements, clinical studies nowadays do not just document clinical efficacy vs. placebo as was 20 years ago, also dose-finding protocols have been established to demonstrate a dose-dependent response for each preparation. Today, treatment effects of allergen immunotherapy (AIT) are assessed by means of symptom severity in patients; however, evaluations of naturally exposed patients do often show weak efficacy [1] due to high individual variability regarding environmental allergen exposure and corresponding symptom burden. Moreover, not only the natural pollen exposure and corresponding symptom severity of the patients are limiting factors for the power of immunotherapy studies, but also the retrospective symptom scoring for baseline evaluation and the combined evaluation of patients with different symptom severity. Allergen challenge test methods, such as conjunctival, nasal, or bronchial challenge tests, or challenges under standardized conditions in allergen exposure chambers (AEC) are accepted and approved by the European Medicines Agency (EMA), but currently limited to phase II (dose-finding and proof-of-concept-) trials [2]. Authorities do not yet accept allergen challenge tests as sole assessment tool for demonstrating efficacy of AIT in pivotal trials. 

This review aims to describe different allergen challenge test methods, summarizes safety and limitations for each, and discusses their potential for use in AIT trials. 

## Nasal allergen challenge 

The clinical relevance of an aeroallergen sensitization is usually determined by allergen challenge testing. In contrast to a single-dose nasal allergen challenge (NAC) providing a yes/no answer, a titrated NAC is an established method to evaluate the reaction threshold in patients with allergic rhinitis. So, the efficacy of antiallergic treatment can be demonstrated in terms of nasal symptom reduction and increase of allergen dose-dependent reaction threshold, respectively. A special value is given in patients with local allergic rhinitis otherwise not being diagnosable [3]. To date, NACs have been used in clinical trials to assess the efficacy of market-approved antihistamines, intranasal corticosteroids, combinations of both, other not yet marketed antiallergic compounds, and AIT products [4, 5, 6]. 

Compared to traditional field trials, NAC protocols can be conducted out of season, under controlled conditions and therefore require fewer participants due to a lower variability of data. They can serve as primary endpoints for preliminary dose-finding and proof-of-concept trials as suggested by the EMA guidelines for AIT product development [2] and for determination of efficacy and safety of AIT preparations. A titrated NAC model was used in a dose-finding study of a house dust mite (HDM) allergoid subcutaneous immunotherapy (SCIT) [7]. 290 HDM-allergic subjects received either one of four active mite allergoid doses (6,667 AUeq/mL, 20,000 AUeq/mL, 50,000 AUeq/mL, or 100,000 AUeq/mL) or placebo over a 12-month study period. It could be demonstrated that the three highest doses decreased the mean symptom scores significantly as compared to placebo, with the 20,000 and 50,000 AUeq/mL doses showing the best efficacy/safety ratio. 

The efficacy and safety of *a Phleum pratense* SCIT was evaluated by Rondón et al. [8] in patients with local allergic rhinitis (LAR). In a 2-year randomized, double-blind placebo-controlled (DBPC) trial, participants received either SCIT for 2 years or placebo for 1 year and SCIT for another year. The study revealed that 83% of participants who had received at least 6 months of SCIT had greater tolerance to higher concentrations of allergen as compared to baseline, and 56% even had negative results to NAC. Another very interesting approach evaluated the clinical efficacy of two human Fel d 1-specific IgG4 antibodies in cat-allergic subjects [9]: in a proof-of-concept study, Orengo et al. [9] challenged 73 cat-allergic participants after they had received a single subcutaneous injection of either 600 mg REGN1908-1909 or placebo, in four NACs (days 8, 29, 57, and 85 after injection) with cat hair. Eight days after a single dose of REGN1908-1909, significant improvements (p= 0.0003) were observed in clinical nasal symptoms. Effects were sustained for the entire 85-day study period. Peak nasal inspiratory flow (PNIF) was also improved by 39% at days 8 and 85, and skin prick test reactivity was decreased by 52% at day 29 as compared to baseline. Placebo and REGN1908-1909 were both well tolerated. 

### Safety and limitations

NAC can be safely applied [10], including to asthmatic patients, as only incorrect technique (inhalation) or very high doses may induce bronchoconstriction. Pharyngeal or ear itching must be expected, but anaphylactic reactions have not been described [10]. Unspecific nasal hyperreactivity can be present in any type of chronic rhinitis and must first be excluded by testing the diluent alone. Diluents have to be unglycerinated, as preservatives contained in e.g. skin prick test solutions, such as glycerol or phenol, are irritative and may produce nonspecific reactions. Furthermore, allergen extracts may not contain the entire allergen profile as present in the native allergenic material and produce false negative results. Finally, in a non-titrated NAC, the amount of allergen applied is usually unphysiologically high compared to the dose inhaled during natural allergen exposure, producing false positive results. 

## Conjunctival allergen challenge 

Conjunctival allergen challenge (CAC) tests are useful in routine clinical practice to determine clinical relevance of a sensitization. Especially in polysensitized patients, it has been shown that CAC reveals reliable results also in allergic rhinitis patients not reporting ocular symptoms [11]. CAC as a research tool is traditionally performed to investigate the response to topical treatments in pharmacological studies or to evaluate the efficacy of AIT [12]. Here, efficacy is usually evaluated by determining the threshold concentration of a titrated CAC before and after AIT. Again, unspecific hyperreactivity must first be excluded by testing the diluent alone in one eye and applying the allergenic solution into the contralateral eye [13] In recent years, CAC was used in a few AIT studies as outcome parameter for clinical efficacy: Kruse et al. [14] summarized data from two double-blind studies in which the participants were actively treated with birch or grass pollen sublingual immunotherapy (SLIT) tablets of different allergen content over a period of 12 weeks. The titrated CAC was assessed before and 4 weeks after treatment, and seasonal combined scores were recorded. It could be shown that 43 out of 87 grass pollen-allergic patients and 70 out of 106 birch pollen-allergic patients lost CAC reactivity. Seasonal rescue medication use (grass pollen SLIT: p = 0.005; birch pollen SLIT: p = 0.025) as well as seasonal symptom and medications scores (grass pollen SLIT: p < 0.001; birch pollen SLIT: p = 0.039) were significantly reduced, and a correlation between the CAC and the seasonal symptom severity could be shown. In a randomized, placebo-controlled phase III trial, Mösges et al. [15] assessed the clinical efficacy of immunotherapy with peptide hydrolysates from *Lolium perenne* grass pollen. Combined symptom/medication scores (CSMS) and a titrated CAC (100, 1,000, 10,000, and 100,000 SQ-U/mL) were recorded before and after treatment. The active group had a significantly lower CSMS (entire pollen period: p = 0.029; peak pollen season: p = 0.041) and a clearly associated reduced CAC reactivity after treatment. In another DBPC phase II dose-finding study, four different cumulative doses (5,100, 14,400, 27,600, and 35,600 SU) of a subcutaneous grass pollen preparation were evaluated by Zielen et al. [16] to determine the optimal cumulative dose in patients with grass pollen-induced allergic rhinitis. Standardized CACs were performed to determine the change in clinical reactivity from baseline to post-treatment total symptom score (TSS) following CAC. All treatment groups (n = 447) showed a statistically significant decrease from baseline in TSS compared to placebo, with the largest decrease observed after 27,600 SU (p < 0.0001). In the studies mentioned here, the efficacy of the applied AIT preparations could be shown not only by reduced subjective symptoms and intra-seasonal CSMS, but also by reduction of conjunctival reactivity. 

### Safety and limitations

CAC can be safely used in clinical practice as well as in research settings, also in patients with dry eye. Unspecific conjunctival hyperreactivity must be expected, is of high intra- and inter-individual variability, and can be quantified with instillation of the diluent as a first step of the CAC. The lack of standardized and validated measures to objectify all symptoms of allergic conjunctivitis limits the applicability of the method to subjective symptom scoring and quantification of conjunctival injection (Figure 1), which was first described in 2003 [17] and further refined consecutively [18]. 

## Bronchial allergen challenge 

In patients with allergic asthma, demonstration of airway hyperresponsiveness (AHR) is a prerequisite for adequate asthma diagnosis [19] and inclusion of asthmatic subjects in clinical AIT trials. To demonstrate efficacy of interventions like AIT, reduction of AHR has to be determined. Here, it has to be differentiated between early (EAR) and late (LAR) asthmatic responses [20] upon bronchial allergen challenge (BAC). In contrast to pollen allergens predominantly inducing an EAR with a FEV_1_ drop within 3 hours upon exposure, perennial allergens like HDM are known to induce LARs with a delay of several hours. 

However, until today, several parameters like subjective symptom scoring of cough, wheezing, and chest tightness or objective measures of FEV_1_, PEF, or FeNO were evaluated in studies [19], but none of these was able to reproducibly reflect EAR and LAR upon BAC. 

Currently, bronchoconstriction is quantified by measuring changes of FEV_1_ after BAC, and a 20% decline of FEV_1_ indicates a positive response. Unfortunately, results of allergen-specific BACs are barely reproducible due to different variables contributing to individual reactivity of the asthmatic patient, e.g., environmental co-exposure with the test allergen, reactivity and exposure to other inhalable allergens, concomitant infections, proper washout of anti-inflammatory medication, etc.... The type of the eliciting allergen may also influence the intensity and time course of the asthmatic reaction. 

Mite-asthmatic subjects were included in a DBPC phase II trial evaluating a mite tablet sublingual preparation in terms of dose finding, onset of action, immediate and long-term efficacy in the Vienna challenge chamber [21]. 124 patients were treated with three different doses versus placebo for 6 months and were assessed by means of systemic environmental mite allergen challenge ahead, after 2, 4, and 6 months of treatment in terms of nasal, ocular, and asthma symptoms. A dose- and time-dependent effect of the SLIT tablet on nasal, ocular, and asthma symptoms was observed, with maximum efficacy of the highest dose at week 24 after 6 months of treatment. It could be shown that higher symptomatic asthmatic patients benefit more from the active treatment. A subset of patients (n = 51) could be re-evaluated 1 year after cessation of treatment. A sustained improvement of symptoms was still evident in the high-dose group [22]. However, subjective asthma symptoms could be objectified in highly sensitized patients only, measuring EARs with FEV_1_ during the allergen challenge and LARs with PEF after the challenge at home, respectively. As LARs are putting the patient at risk of losing asthma control several hours after the BAC, an asthma action plan was established and maintained in this study, which is advisable for all allergen challenge studies evaluating efficacy with regards to asthmatic reaction. 

### Safety and limitations

BAC is safe when conducted in a study setting with experienced staff. Asthma exacerbations and LAR may occur. Therefore, it is obligatory that during and after the challenge an asthma action plan including rescue medication is always available. Until today, BAC is used by only a small number of research institutions. The use of BAC in AIT studies is limited by the need for adequate washout of any antiasthmatic medication, which is in conflict with maintaining asthma control. The variability of clinical reactivity of the study participants, which is impacted by a number of personal and environmental factors, and the lack of validated objective parameters to document AHR in general as well as EAR and LAR, respectively, are further contributing to our current challenges of successfully demonstrating the added value of AIT for the management of asthma. Limitations regarding allergenic material are the same as for NAC. 

## Allergen exposure chambers 

Allergen exposure chambers (AECs) were developed as a research tool in the 1980s in order to evaluate pollen-allergic patients under controlled and reproducible pollen exposure conditions independent of natural environmental exposure. AECs have been used for evaluation of allergen-specific immunotherapy since the late 1990s, and the AEC model was approved and accepted by FDA and EMA for phase II immunotherapy trials including proof-of-concept, dose determination, onset and duration of action protocols as well as tolerability and safety and for further supporting phase III trials. 

**Figure 2:**
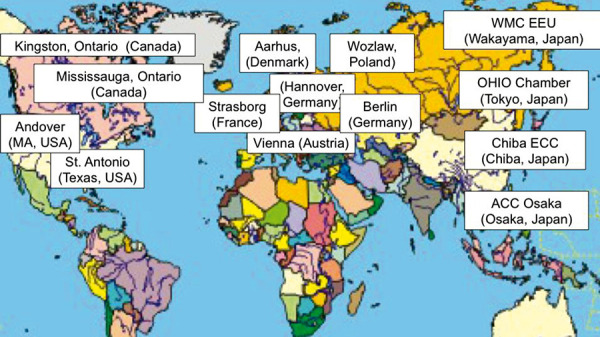
Overview of currently operative allergen exposure chambers [24].

Today, AEC models for grass and ragweed pollen, tree pollen, Japanese cedar pollen, HDM, and cat epithelia exist, and AECs are available in Europe (Germany, Austria, Belgium, France, Poland), North America (USA and Canada), and Asia (Japan) [23] (Figure 2). 

In contrast to organ-specific allergen challenge test methods, the systemic allergen exposure in an AEC targets all organs affected by inhalable allergens (eyes, upper and lower respiratory tract) simultaneously. Depending on the study design, allergic rhinitis, conjunctival and bronchial symptoms are evaluated in regular intervals throughout the challenge session and objectified by different assessments if validated methods are available. For example, the symptom “rhinorrhea” can be quantified by weighing nasal secretions, or “nasal obstruction” by means of nasal air flow measurement. During the past decades, a broad range of AIT preparations was investigated in AEC studies: one of the first AIT studies conducted in an AEC evaluated the efficacy and tolerability of a short-term birch pollen SLIT [25]. 41 birch pollen-allergic subjects were treated in this DBPC study for 4 months and evaluated clinically and immunologically before and 4 months after treatment. Titrated skin prick tests and CAC were carried out to determine the threshold of reactivity. During a 2-hour birch pollen challenge in the VCC, subjective nasal, conjunctival, and bronchial symptoms were documented via a visual analog scale; nasal congestion, nasal secretion, and bronchial reactions were objectified. A significant effect of the active treatment could be demonstrated in all subjective and objective clinical assessments. A very early onset of action within 1 week of treatment was shown in the first published grass pollen immunotherapy study conducted in an AEC [26]. 89 allergic rhinitis patients were randomized to receive a 300-IR active or placebo tablet and were assessed in the VCC at baseline, after 1 week, 1 month, 2 months, and 4 months of treatment. First treatment effects on nasal and ocular symptoms were detectable after 1 week, and statistically significant efficacy could be shown after 1 month. A very interesting other study investigated the induction of cross-tolerance of a grass SLIT tablet on birch pollen-induced allergic rhinitis symptoms [27]. After 4 months of treatment, symptom scores of the 87 subjects included in the efficacy analysis were comparable in the SLIT group and the placebo group, indicating that treatment effects are allergen specific only with no bystander effects. 

### Safety and limitations

Symptoms of allergic rhinoconjunctivitis and/or early asthmatic reactions induced in an AEC either resolve spontaneously or can easily be treated with antiallergic and bronchodilator medication after the end of the session. As late asthmatic reactions may occur, especially after challenge with perennial allergens like cat or mite, it is obligatory to develop and maintain an asthma action plan including rescue medication as already mentioned for BAC. Currently, challenge chamber systems and evaluations done in there are very heterogenous, and comparability of data generated in different AECs is limited. 

So, some allergen models are standardized on the allergen content dispersed and others are standardized on a target symptom level to be induced. A dose-response relation could not be determined for all models. The duration of exposure depends on the data sets to be evaluated. Usually, at least the area under the curve over a minimum of 2 hours of a stable symptom plateau reaction is calculated. Some investigators also determine the late-phase allergic reaction after several hours of continuous allergen exposure and study the effect of repetitive AEC exposures of a series of consecutive days. The use of AECs for assessment of clinical efficacy of different immunotherapeutic preparations in phase III pivotal trials is still limited by the operational availability of validated systems and allergen models and by regulatory restrictions driven by the claim to demonstrate the comparability of treatment effects under natural environmental exposure with AEC exposure conditions [28]. 

## Discussion and outlook 

Assessment of patients with airway allergies for evaluation of new treatment compounds and pathophysiological disease mechanisms is tricky for several reasons: The development of measurable allergic symptoms is depending on exposure to the disease-eliciting allergen source, and the course of the clinical disease is impacted by environmental and immunological factors, of which not all are known yet. To generate statistically evaluable data for dose-finding, efficacy, or even duration of action of an AIT investigational product, large numbers of patients and long observation periods are necessary. Moreover, variable allergen exposure and other environmental factors could lead to unexpected insignificant results in field studies. Organ-specific allergen challenges like nasal, conjunctival, or bronchial provocation tests provide information about the individual clinical reactivity and reaction threshold as well as organ-specific efficacy of a treatment compound applied. However, the results of these tests cannot always be extrapolated to other organ systems and are potentially complicated by side effects due to unphysiologically high allergen concentrations. To overcome the disadvantages of these trial designs, the first AEC model was developed in Vienna in the 1980s to investigate disease mechanisms and treatment effects under controlled and reproducible conditions. Meanwhile, AECs are available worldwide and increasingly recommended by regulatory authorities for investigation of new compounds due to the high quality of the data obtained and the efficiency of these systems in terms of costs and time. AEC study participants are traditionally examined in a symptom-free interval out of season to allow for omission of antiallergic concomitant medication, which would compromise study results, before and during the AEC session. In field studies, conducted under natural exposure conditions, an interruption of the concomitant medication is not feasible for ethical reasons. Due to a considerable reduction of variables impacting study results, AEC studies require significantly less time and smaller numbers of individuals and have the potential to reduce the cost of AIT development substantially. 

One of the primary future needs will be the harmonization of the various challenge protocols, which differ in their validation and standardization, and the correlation with data derived from natural exposure evaluations. In AEC systems, the evidence concerning validity and reliability is steadily increasing, showing comparability of AEC models, reproducibility of clinical effects at different time points, and possible extrapolation to natural exposure of patients. 

Currently, there are protocols in place for standardized nasal, conjunctival, and (unspecific) bronchial challenge in clinical routine for diagnostic and monitoring purposes, which are safe and kept as efficient as possible for economic reasons. For clinical research needs, more than just one validated parameter is usually measured, as for documentation of clinical and immunological performance of a novel AIT product quite a lot of data is required. Reproducibility and a narrow range of variability are crucial, but maximizing patients´ symptoms by exposure to high allergen concentrations leads to overestimation of treatment effects in phase II trials with the consequence that consecutive phase III field trials very often fail: due to a low symptom level elicited by low environmental allergen concentrations, e.g., during weak pollen seasons, a lack of discrimination between active and placebo may then occur. So, if using challenge testing as a measure to determine efficacy of an AIT preparation, the allergen concentration should be as physiological as possible. 

## Conclusion 

A high level of standardization with a sufficient toolbox of validated parameters has been established globally for NAC, but not yet achieved for the other challenge methods: in CAC and BAC the toolbox is limited to subjective symptom scoring, as no suitable and validated objective parameters have been identified so far. The setup of an AEC is very complex, and systems are still heterogenous; here, harmonization or at least correlation of results retrieved is required to enable comparability of study data. 

All challenge methods can be safely applied when provided and monitored by skilled and experienced staff. 

## Funding 

None. 

## Conflict of interest 

PZ: has received lecture fees from Alk Abello, Allergopharma, Allergy Therapeutics, HAL, Leti, Meda, Merck, Novartis, Stallergenes, Thermo Fisher Scientific, scientific and educational grants from Alk Abello, Allergopharma, Allergy Therapeutics, Biomay, Calistoga, GSK, HAL, Marinomed, MSD, Ono, Oxagen, RespiVert, Stallergenes, VentirX, and is board member of Alk Abello, Bencard, Meda, Merck, Novartis, Sigmapharm, and Stallergenes. RZ: none, PL: none. 

**Figure 1:**
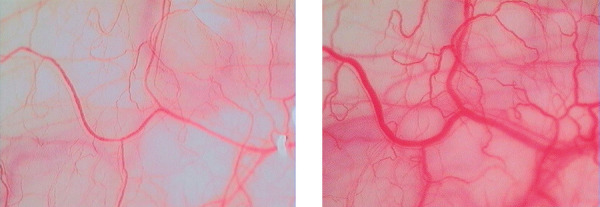
Conjunctival injection before (left) and after (right) a 4-hour grass pollen challenge in the Vienna challenge chamber.
